# Kettlebell Training vs. Plyometric Training: A Comparison of Jump Performance in Volleyball and Basketball Athletes

**DOI:** 10.3390/jfmk10040395

**Published:** 2025-10-12

**Authors:** Tom Brandt, Lucas Koch, Maximilian Herber, David Ohlendorf, Annette Schmidt

**Affiliations:** 1 Sports Biology, University of the Bundeswehr Munich, 85579 Neubiberg, Germanymaximilian.herber@unibw.de (M.H.);; 2 Kompetenzzentrum für Funktionsfitness, University of the Bundeswehr Munich, 85579 Neubiberg, Germany

**Keywords:** jump height, vertical jump, countermovement jump, squat jump, drop jump

## Abstract

**Objectives:** Plyometric training is a well-established method for enhancing jump performance in basketball and volleyball athletes but has certain limitations. Kettlebell training may provide a viable alternative as it mimics key biomechanical aspects of jumping, like explosive hip and knee extension during a ballistic hip–hinge pattern. Because evidence remains limited, this study aimed to compare the effects of both training methods. **Methods:** Thirty-eight volleyball and basketball club athletes (age: 22 (4.3); male = 29, female = 9) completed this study. Countermovement jump (CMJ), squat jump (SJ), drop jump (DJ), body fat percentage (FM), and muscle mass percentage (MM) were assessed pre- and post-intervention. The participants were assigned to one of three groups: a kettlebell training group (KbG), a plyometric training group (PG), or a control group (CG). Both the KbG and PG completed two supervised 25-min training sessions per week for six weeks, while the CG did not engage in any additional training intervention. The level of significance was set at *p* ≤ 0.05. **Results:** There were no significant differences in CMJ, SJ, and DJ performance between the groups before the intervention. Significant differences in change between the groups from pre- to post-test were found for the SJ (*p* = 0.006), but not for the DJ (*p* = 0.06), CMJ (*p* = 0.26), FM (*p* = 0.9), and MM (*p* = 0.55). Pairwise comparisons revealed significantly greater positive change in the KbG than in the CG for the SJ (*p* = 0.003) and DJ (*p* = 0.03). Within-group analyses showed significant improvements in the KbG for the CMJ (*p* = 0.04), SJ (*p* < 0.001), and DJ (*p* = 0.003) performance, whereas FM and MM did not change. Within the PG and CG, no significant change occurred. **Conclusions:** Kettlebell training effectively improved jump performance and may therefore serve as a valuable component within strength and conditioning programs for basketball and volleyball athletes.

## 1. Introduction

Sports such as basketball and volleyball are characterized by explosive and high-velocity movements, with jump performance closely linked to in-game performance. Accordingly, jump training constitutes a central component of the strength and conditioning programs for athletes in these disciplines [1,2,3,4]. Plyometric training is a well-established method for enhancing jump performance, primarily by targeting the stretch–shortening cycle (SSC) [3,5]. The SSC involves a rapid eccentric muscle action immediately followed by a concentric contraction of the same muscle group, thereby stimulating the neuromuscular system and enhancing coordination [5,6]. Despite its effectiveness, plyometric training also presents certain limitations. It places high mechanical stress on joints, is often performed at high intensities, and can lead to acute muscle soreness, which may increase the risk of musculoskeletal injuries [7,8]. Given that many sport-specific training programs in basketball and volleyball already incorporate SSC elements, such as jumps, rapid directional changes, and explosive starts, alternative training modalities may be beneficial [3].

Kettlebell training represents one such alternative. Exercises like kettlebell swings mimic key biomechanical aspects of jumping, like explosive hip and knee extension during a ballistic hip–hinge pattern, strongly engaging the posterior chain. They also emphasize rapid eccentric control during the SSC [9]. Beyond swings, kettlebells can also be used to load squat movements (e.g., goblet squats) as well as unilateral movements (e.g., lunges, split squats). When performed with maximal concentric acceleration, explosive kettlebell goblet squats could resemble the loading pattern of squat jumps, yet without a flight phase, thereby emphasizing vertical force production under load and providing an effective stimulus to improve jump performance [10,11]. It is therefore plausible that neuromuscular and morphological adaptations induced by kettlebell training could also enhance jump performance [10,12,13,14,15].

Nevertheless, previous studies investigating the effects of kettlebell training on jump-related performance parameters (e.g., countermovement jump (CMJ), squat jump (SJ), and drop jump (DJ)) have yielded mixed results, with some reporting significant improvements and others finding no notable changes. These interventions typically employed a training frequency of two or three sessions per week over a period of 6 to 12 weeks [10,12,13,14,15,16].

However, no studies have compared the effects of kettlebell and plyometric training on jump performance in basketball or volleyball athletes. The aim of this study was therefore to examine and contrast these two types of training against a control condition on key jump performance parameters. It was hypothesized that both interventions would improve jump performance compared with the control group, but no significant differences were expected between the two types of training.

## 2. Methods

### 2.1. Experimental Approach to the Problem

This study was conducted as a non-randomized controlled trial with three groups: a kettlebell training group (KbG), a plyometric training group (PG), and a control group (CG). The intervention was conducted at the University of the Bundeswehr Munich (UniBw M). The participants underwent standardized performance assessments at baseline (pre-test) and following a six-week intervention period (post-test). Group allocation was based on self-selection. While the participants in the KbG and PG completed a structured 6-week training program specific to their group, consisting of two weekly sessions of 25 minutes [min] each, the CG was instructed to maintain their habitual physical activity levels throughout the study. Compliance within the CG was monitored using a self-report questionnaire.

### 2.2. Subjects

Thirty-two volleyball (KbG: *n* = 15, PG: *n* = 9, CG: *n* = 8) and 6 basketball (KbG: *n* = 1, PG: *n* = 1, CG: *n* = 4) athletes with a minimum of 1 year of continuous training experience in a club setting were eligible for inclusion. The exclusion criteria comprised the following: (a) pregnancy, (b) chronic or acute medical conditions contraindicating participation in physical exercise or performance testing (e.g., severe musculoskeletal injuries, osteoporosis, intervertebral disc pathology, joint replacements, hypertension, or recent surgical scars), (c) engagement in additional structured training programs beyond regular club activities during the intervention period, (d) attendance at fewer than 80% of the prescribed kettlebell or plyometric training sessions, and (e) current or previous use of anabolic steroids. The inclusion and exclusion criteria were assessed via a standardized questionnaire administered at both pre- and post-intervention testing. According to an a priori power analysis, 12 participants per group were determined to achieve a power of 80% on a one-sided, 5% significance level. An overview of the demographic and anthropometric characteristics of the participants is presented in Table 1.

The Institutional Ethics Committee of the UniBw M approved the study protocol, ensuring that it conformed to the ethical guidelines of the 1975 Declaration of Helsinki. Informed consent was obtained from all subjects involved in the study (18 November 2024; EK UniBw M 24–60).

### 2.3. Procedures

#### 2.3.1. Training Interventions

The KbG and PG trained twice per week for 25 min in advance of their regular sport-specific club training. All sessions were conducted in a group setting under the supervision of a coach to ensure correct form and adherence to the training protocol. Between training sessions, there were at least 48 h of rest. Before the 6-week intervention program, the KbG and PG participated in a familiarization session. This session was meant to introduce the exercises and determine the respective training loads the participants were able to move with correct form.

##### Kettlebell Training

The kettlebell training was conducted at the UniBw M. Every training week included 2 training sessions. Each session started with a 5-min dynamic warmup. Based on previous research, kettlebell swings and goblet squats appear to be effective exercises to improve jump height and were therefore included. Lunges, as a unilateral exercise, were added to the program to account for potential unilateral weaknesses [10,11,14]. Since the comparison of the studies by Lake and Lauder, as well as Otto et al., indicated that a higher kettlebell training volume was more effective, the number of repetitions was increased compared to the study by Otto et al. [14]. In the first weekly session, the participants completed Russian kettlebell swings followed by goblet squats (each exercise for 5 sets of 12 repetitions), with 40 seconds [s] of rest between sets and exercises. For the Russian kettlebell swing, the participants were instructed to execute the concentric portion as explosively as possible with full extension at the hips. The goblet squats were performed with a controlled eccentric (1.5 s) and an explosive concentric phase (<1 s). During the second weekly session, the participants performed Russian kettlebell swings again for 5 sets of 12 repetitions and 40 s rest between sets. Afterward, they performed kettlebell front rack lunges for 5 sets of 12 repetitions per leg, switching legs after each set, with 40 s of rest in between. While the eccentric part of the lunges was meant to be completed in a controlled manner (1.5 s), with the knee stopping 1 centimeter [cm] above the ground, the concentric motion was intended to be performed explosively (<1 s). After completion of week 3, the training loads of each movement were increased by 4 kilograms [kg].

##### Plyometric Training

The plyometric training was carried out twice per week in a sports hall with synthetic sports flooring. The sessions started with a 5 min dynamic warm-up. Based on previous research, each session included more than 50 jumps and incorporated a variety of jump types [17,18]. The first weekly workout session consisted of pogo jumps, a 4-square drill, and drop jumps from a 20 cm height. In the second weekly session, the participants performed single-leg line jumps, jumping lunges, and box jumps. All exercises were performed for 3 sets of 6 repetitions with 2 min of rest between sets. In the single-leg line jump, each set consisted of 6 jumps per leg. For the plyometric movements, the participants aimed for maximum height and short ground contact times.

#### 2.3.2. Test Protocol and Outcomes

The pre- and post-tests were carried out by the same personnel who conducted the training sessions. Neither the test personnel nor the participants were blinded. The participants had to avoid any intense physical activity 24 hours [h] before the test sessions. They were further asked to maintain the same fluid and food intake on both test days. The testing started with a questionnaire, including questions regarding demographic data and exclusion criteria. Next, height and body composition were measured in underwear. Height was assessed with a SECA^®^ 213 (seca GmbH & Co. KG, Hamburg, Germany) and body composition with a SECA^®^ mBCA 515 scale (seca GmbH & Co. KG, Hamburg, Germany) [19].

Before the performance tests, the participants underwent a supervised warm-up program, including 2 min of running in place, 1 min of jumping jacks, 30 s of knee raises in place, 30 s of butt kicks, 30 s of skipping, 10 bodyweight squats, and 5 burpees. The tests were performed on dual force plates (TEMPLO^®^ Performance Analysis Contemplas GmbH, Kempten, Germany) and analyzed using Templo performance analysis software version 2023.0.754 (TEMPLO^®^ Performance Analysis Contemplas GmbH, Kempten, Germany) to assess maximal vertical jump height (given in cm) in the squat jump (SJ), countermovement jump (CMJ), and drop jump (DJ) from 20 cm height. For the actual testing, each jump was executed 3 times for maximum height with 60 s rest between jumps.

The main outcomes of interest were changes in jump performance (CMJ, SJ, and DJ) from pre- to post-test, considering both between- and within-group differences. In addition, changes in body fat percentage (FM) and muscle mass percentage (MM) were examined as exploratory outcomes.

### 2.4. Statistical Analyses

The normal distribution was examined via Q-Q plots and the Shapiro–Wilk test. To analyze differences in change between the groups, change scores were computed for CMJ, SJ, DJ, FM, and MM by subtracting the pre- from the post-test value. Baseline differences between the groups were evaluated with the Kruskal–Wallis test. Differences in change scores were likewise analyzed via the Kruskal–Wallis test. Post hoc pairwise comparisons of the KbG vs. CG and the PG vs. CG were tested one-tailed (KbG > CG and PG > CG) using the Mann–Whitney-U test, whereas the KbG vs. PG, as well as pairwise tests for FM and MM, were two-tailed. To account for multiple testing, the Bonferroni correction was applied. Within-group changes from pre- to post-test were analyzed using the Wilcoxon signed-rank test (one-tailed for the KbG and PG with post > pre and two-tailed for the CG). Values are presented as median (interquartile range (IQR)). Statistical significance was set at *p* ≤ 0.05. Effect sizes are given as rank epsilon-squared (ε^2^_H_) and computed based on the sample size, the number of groups, and the tie-corrected H‘ statistic when the Kruskal–Wallis test was applied. The effect size r was calculated based on the z-value and the sample size when the Wilcoxon signed-rank test and the Mann–Whitney-U test were used. Data analysis was performed with SPSS version 29.0.2.0 and JASP version 0.95.

## 3. Results

**Figure 1:**
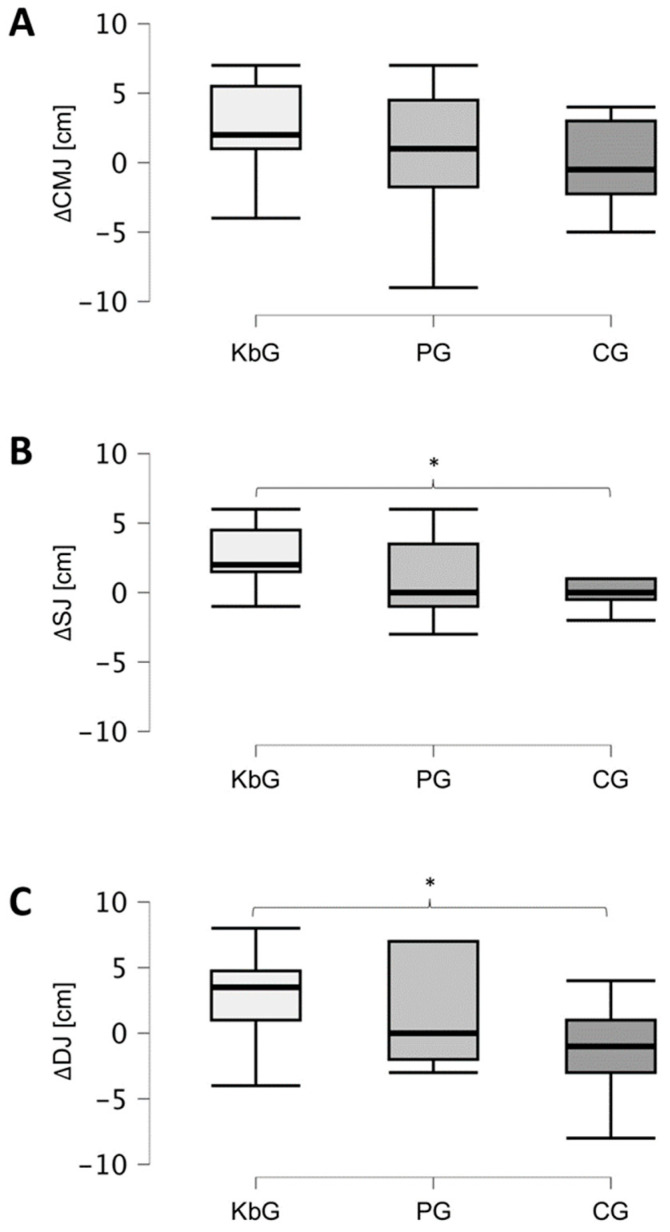
Differences in countermovement (**A**), squat (**B**), and drop (**C**) jump performance between pre- and post-test. Abbreviations: KbG = kettlebell training group; PG = plyometric training group; CG = control group; ΔCMJ = countermovement jump difference between pre- and post-test; ΔSJ = squat jump difference between pre- and post-test; ΔDJ = drop jump difference between pre- and post-test; cm = centimeter, * significant difference for pairwise comparison (*p* < 0.05).

There were no significant differences in the CMJ (*p* = 0.21), SJ (*p* = 0.43), DJ (*p* = 0.24), FM (*p* = 0.13), and MM (*p* = 0.29) in the pre-test between the groups. Significant differences in change scores between the groups from pre- to post-test were found for SJ (*p* = 0.006), but not for DJ (*p* = 0.06), CMJ (*p* = 0.26), FM (*p* = 0.9), and MM (*p* = 0.55). Pairwise comparisons showed significantly greater positive change in the KbG than in the CG for SJ (*p* = 0.003) and DJ (*p* = 0.03). The change scores are further illustrated in Figure 1.

While the PG and CG showed no significant within-group changes, the KbG demonstrated significant positive changes in the CMJ (*p* = 0.04), SJ (*p* < 0.001), and DJ (*p* = 0.003). Analyses of changes in FM and MM revealed no significant differences in any group. The pre- and post-measures, as well as effect sizes and *p*-values, are presented in Table 2.

## 4. Discussion

This 6-week intervention study aimed to investigate the effects of kettlebell and plyometric training on jump performance parameters in experienced, recreational basketball and volleyball athletes. Between-group analyses revealed a significant effect only for the SJ, with pairwise comparisons showing greater improvements in the KbG compared with the CG. While no overall between-group effect was detected for the DJ, pairwise comparisons indicated that the KbG improved significantly more than the CG. Furthermore, the results demonstrated significant within-group improvements in CMJ, SJ, and DJ performance for the KbG, whereas no significant changes were observed in the PG or CG.

In line with previous research, these results suggest that the mechanical demands of kettlebell training may provide an effective stimulus to improve jump performance in athletes already accustomed to jump-specific sport movements [11]. Nevertheless, the jump height improvements observed in the KbG (CMJ: 3.9%) were still lower than the mean change (CMJ: 15%) reported after a 6-week intervention study conducted by Lake and Lauder [10]. However, the present study exclusively involved experienced basketball and volleyball athletes, reinforcing the idea that prior exposure to jump-specific training may substantially modulate the magnitude of performance adaptations. Additionally, a longer kettlebell intervention over five months in female ballet dancers led to a 39.16% improvement in jump performance, further underscoring the critical role of intervention duration in achieving meaningful performance adaptations [12]. Contrastingly, despite identical intervention duration and training frequency, as well as similar exercise selection, the improvements in the present study exceeded those reported by Otto et al. (2.2%). Notably, Otto et al. ensured progressive overload by increasing training volume, while in the present study, the training load was increased (+4 kg after 3 weeks of training) [14]. Moreover, when comparing the training protocols of the present study with the 6-week interventions of Lake and Lauder, as well as Otto et al., notable differences in training volume become apparent. Lake and Lauder prescribed 24 sets per week, each consisting of 30 s kettlebell swings followed by 30 s rest, which—estimating on average 19 repetitions per round based on the findings of Budnar et al.—corresponding to 456 repetitions per week. Contrastingly, the present study involved 20 sets per week (including kettlebell swings, goblet squats, and lunges; 240 repetitions), and Otto et al. reported 22 (week 1–3) and 28 sets (week 4–6), corresponding to 116 and 144 repetitions [10,14,20]. This comparison suggests that the overall number of repetitions may represent a decisive factor for improvements in jump height. The greater improvement in SJ performance in the KbG may partly be explained by the inclusion of explosive goblet squats and lunges, both emphasizing rapid concentric force production, as well as the close similarity in movement execution between the goblet squat and the SJ. In combination with the posterior chain engagement and explosive hip extension required during kettlebell swings, this set of exercises might have provided an appropriate stimulus for enhancing SJ performance. Despite these positive changes, it remains unclear whether the magnitude of improvements in jump height was of practical relevance in real in-game situations.

The importance of specificity has also been noted in plyometric training research, indicating that the specific contraction types of movements performed during training lead to differing adaptations in fast SSC jumps (e.g., DJ), slow SSC jumps (e.g., CMJ), and jumps involving solely a concentric muscle action (e.g., SJ) [5]. Despite the good specificity of the exercises performed during the plyometric training sessions, changes in the CMJ, SJ, and DJ were neither statistically significant in the within- and between-group analyses, nor did they reach the average increase of 7%, respectively, or 3.9 cm, that Villarreal reported for plyometric training interventions [5]. Notably, Azreh et al. even reported CMJ improvements of 20.3% following a 6-week plyometric training intervention. However, their study explicitly excluded individuals with a background in basketball or volleyball, which suggests that the pronounced gains were likely driven by a “beginner effect” [21]. Conversely, the participants in the present study had prior experience in jump-dominant sports, which may have attenuated their responsiveness to additional plyometric stimuli. Moreover, in contrast to the KbG, the PG performed fewer repetitions per week and did not incorporate progressive overload, which could have reduced the effectiveness of the training stimulus. When interpreting the limited responsiveness of the PG, the intervention duration needs to be considered. Evidence suggests that plyometric training durations shorter than 10 weeks yield only small improvements in jump height [22]. This aligns with the present study’s effects for plyometric training, which fall below the pooled effect size reported in a meta-analysis by Makaruk et al. (SMD = 0.68; 95% CI [0.37, 0.99]) [23]. Another meta-analysis focusing exclusively on female participants supports these findings, showing substantially larger effect sizes for long-term interventions (≥ 12 weeks) [24].

Similar to the 6-week intervention study by Otto et al., who compared kettlebell training with weightlifting over the course of 6 weeks, no significant change in body composition was found in any group, which could be explained by the low amount of additional training volume and short intervention duration. However, this also indicates that performance improvements and trends in the KbG and PG were primarily driven by neural adaptations [14].

In addition to its effectiveness, kettlebell training proved highly feasible and well-suited for application in group settings. Only minimal space and equipment were required, and the simplicity of the movements allowed the participants to develop sufficient technical proficiency within the 6-week period to perform the exercises safely and independently. While this highlights the low coaching demand, a familiarization phase is recommended to establish safe and effective technique before progressing to more advanced or load-intensive variations. To sustain long-term engagement, it is advisable to incorporate new exercises periodically. The wide variety of available kettlebell movements enables high levels of training variation, which can help maintain athlete motivation and address diverse performance goals over time.

Despite the insights gained from this study, several limitations should be acknowledged. First, the non-randomized allocation of the participants to the groups introduced a potential selection bias, which may have influenced group characteristics beyond the measured variables and limited internal validity. Second, while the overall sample size was determined through an a priori power analysis, the relatively small and uneven number of participants per group limits the generalizability of the findings. This issue was particularly pronounced in the PG, leading to reduced statistical power and less robust analyses. Another limitation of this study is the unbalanced distribution of sex (29 men vs. 9 women) and sport (32 volleyball vs. 6 basketball) within and across groups. This imbalance may have influenced the outcomes, as sex-specific differences as well as different training loads can not be ruled out. Due to the limited sample size, stratified analyses by sex or sport were not conducted. Furthermore, neither the participants nor the researchers were blinded, potentially increasing susceptibility to bias. Moreover, the short intervention duration of six weeks may not have been sufficient to elicit maximal physiological adaptations, particularly in trained individuals. In addition, the kettlebell group accumulated a greater training volume and incorporated progressive overload, whereas the plyometric group performed fewer repetitions without systematic progression. This imbalance may have contributed to the smaller effects in the PG. Additionally, whether additional resistance training was performed at the clubs was not systematically monitored. Finally, while a familiarization session was conducted before the intervention period, the extent to which the participants mastered each movement pattern may have varied individually.

Considering these limitations, future studies should employ randomized controlled designs, recruit larger and more balanced samples in terms of sex and sport, and extend the intervention duration. Beyond methodological improvements, it is further recommended to systematically examine program variables, such as volume, frequency, exercise type, and intensity, to optimize the efficacy of kettlebell and plyometric training interventions. In particular, future work should address (a) the optimal intervention duration for experienced athletes, (b) the comparative benefits of different kettlebell exercises on distinct jump parameters, (c) interactions between training frequency and load intensity, and (d) the specific biomechanical and neuromuscular mechanisms underlying observed performance improvements. Lastly, future research should evaluate the feasibility and effectiveness of kettlebell and plyometric training in other populations that rely on strength and power, such as military personnel, with particular emphasis on female soldiers.

### Practical Applications

Kettlebell training could be applied to improve jump-related performance parameters relying on explosive and reactive strength due to its potential to induce comprehensive neuromuscular adaptations. From a practical standpoint, kettlebell training is an accessible and low-barrier training option as it requires minimal equipment, is cost-efficient, and can be performed in limited space. This makes it ideal for implementation during in-season periods when training load must be managed or in settings with limited access to high-tech facilities. Furthermore, kettlebell training may be particularly valuable for athletes with prior exposure to plyometric training, offering novel stimuli while reducing joint strain. Coaches as well as strength and conditioning practitioners are encouraged to integrate kettlebell training strategically, either as a stand-alone program during off-season preparation phases or as a supplemental modality during maintenance and recovery phases. Kettlebell training may also serve as a low-impact alternative for athletes recovering from injury or dealing with chronic joint issues, allowing continued power development without excessive stress on vulnerable structures.

## 6. Conclusions

This study showed that short-term kettlebell training interventions could improve jump performance in recreational volleyball and basketball athletes. While it remains unclear whether these improvements are of practical relevance in real in-game contexts, the applied training program, consisting of kettlebell swings, goblet squats, and lunges performed twice per week, should still be considered as an alternative to plyometric training. Concluding, kettlebell training can be considered as a practical, versatile, and effective training tool for improving jump performance in sport-specific training environments.

## Figures and Tables

**Table 1:** The subjects’ characteristics.

Variable	Total Sample (*n* = 38)	Kettlebell Intervention (*n* = 16)	Plyometric Intervention (*n* = 10)	Control (*n* = 12)	*p*-Value
Sex (male/female)	(29/9)	(13/3)	(9/1)	(7/5)	0.216
Age [yrs]	22 (4.3)	22 (3.8)	21 (7)	22 (4.8)	0.463
Height [cm]	181.5 (12.3)	180 (12.5)	183.5 (17)	176 (21)	0.304
Weight [kg]	74.6 (17.1)	73.6 (16.8)	71.9 (17)	76.9 (20.8)	0.935
BMI [kg/m^2^]	23.63 (3.9)	23.7 (2.8)	22 (3.9)	24.6 (4.9)	0.159
FM [%]	17 (11.2)	16.3 (12.7)	15.4 (5.7)	17.6 (12.2)	0.129
MM [%]	41.3 (4.6)	41.3 (5.4)	41.3 (3.1)	39.9 (9.4)	0.293

Note: Values are presented as median (interquartile range (IQR)). The significance level was set at *p* < 0.05. Abbreviations: BMI = body mass index; FM = fat mass percentage; MM = muscle mass percentage; yrs = years.

**Table 2:** Change within and between groups from pre- to post-test.

	Pre-Test	Post-Test	Change Score	Change Within Groups		Difference in Change Between Groups	
				r	*p*	ε^2^_H_	*p*
CMJ [cm]						0.02	0.26
KbG	41.5 (17.5)	44 (13.5)	1.5 (5.5)	0.45	0.04		
PG	42 (8.5)	45.5 (13.8)	1 (7.3)	0.21	0.25		
CG	35.5 (17.8)	35.5 (21)	−0.5 (5.8)	0.01	0.96		
SJ [cm]						0.24	0.006
KbG	36 (9)	35.5 (10.8)	2 (4.5)	0.81	<0.001		
PG	36.5 (10.3)	38 (8.3)	0 (5.3)	0.29	0.18		
CG	32 (17.5)	31 (16)	0 (2.5)	0.12	0.67		
DJ [cm]						0.12	0.06
KbG	30 (11)	34 (8.8)	4 (5)	0.7	0.003		
PG	29 (9)	29.5 (6.3)	0 (9.5)	0.38	0.13		
CG	21 (17.5)	17 (16)	−1 (7.5)	0.24	0.48		
FM [%]						−0.05	0.90
KbG	16.3 (12.7)	16.5 (10.6)	−0.03 (2.4)	0.08	0.76		
PG	15.4 (5.7)	15.6 (7.2)	0.2 (1.3)	0.08	0.8		
CG	17.6 (12.2)	20.4 (11.6)	0.1 (1.6)	0.05	0.88		
MM [%]						−0.02	0.55
KbG	41.3 (5.4)	41 (5.3)	−0.3 (1.2)	0.13	0.61		
PG	41.3 (3.1)	41 (2.7)	−0.4 (1.7)	0.24	0.45		
CG	39.9 (9.4)	39.5 (8.9)	0.1 (1.2)	0.16	0.58		

Note: The change score was computed by subtracting the pre-test value from the post-test value. Values are presented as median (interquartile range (IQR)). For analyses of within-group changes, the effect size r is reported. Rank epsilon-squared is given for the between-group analyses of change scores. Significance level was set at *p* < 0.05. Abbreviations: CMJ = countermovement jump; DJ = drop jump; FM = fat mass percentage; MM = muscle mass percentage; SJ = squat jump; ε^2^_H_ = rank epsilon-squared; r = effect size r.

## Data Availability

The data that support the findings of this study are available from the corresponding author upon reasonable request.
